# Intraocular pressure effect of intravitreal conbercept injection for retinopathy of prematurity

**DOI:** 10.3389/fphar.2023.1165356

**Published:** 2023-05-30

**Authors:** Caifeng Gao, Ge Mu, Huanhuan Zhao, Jiao Zheng, Qingyang Feng, Yining Wu, Yinan Li, Xuelin Huang, Wei Sun

**Affiliations:** ^1^ Department of Ophthalmology, Guangdong Women and Children Hospital, Guangzhou, China; ^2^ Department of Anesthesiology, The First Affiliated Hospital of Guangzhou Medical University, Guangzhou, China; ^3^ Department of Ophthalmology, Guangdong Eye Institute, Guangdong Provincial People’s Hospital (Guangdong Academy of Medical Sciences), Southern Medical University, Guangzhou, China

**Keywords:** retinopathy of prematurity, anti-VEGF, conbercept, intraocular pressure, intravitreal injection

## Abstract

**Purpose:** Intravitreal injection of conbercept (IVC) is a novel anti-vascular endothelial growth factor (anti-VEGF) treatment for retinopathy of prematurity (ROP). This study aimed to assess the intraocular pressure (IOP) effect of IVC.

**Methods:** All IVC surgeries were performed in the Department of Ophthalmology, Guangdong Women and Children Hospital, from January 2021 to May 2021. In this study, 30 eyes of 15 infants who received intravitreal injections of conbercept at a dose of 0.25 mg/0.025 mL were included. The IOP of all participants was measured prior to administering the injection and subsequently at 2 min, 1 h, 1 day, and 1 week thereafter.

**Results:** We included 30 eyes (10 boys and 5 girls) with ROP. For the male group, the mean birth weight, mean gestational age at birth, and the mean time of postmenstrual age (PMA) at IVC treatment were 1,174.0 ± 446.0 g, 28.4 ± 3.0 weeks, and 37.1 ± 1.6 weeks, respectively; for the female group, they were 1,108 ± 285.5 g, 28.2 ± 2.5 weeks, and 36.8 ± 2.1 weeks, respectively. For the male group, the IOP at baseline, 2 min, 1 h, 1 day, and 1 week after IVC were 12.4 ± 1.5 mmHg, 49.0 ± 3.1 mmHg, 26.3 ± 2.5 mmHg, 13.4 ± 2.2 mmHg, and 11.6 ± 1.7 mmHg, respectively; for the female group, they were 10.7 ± 2.0 mmHg, 47.3 ± 3.2 mmHg, 26.4 ± 3.2 mmHg, 10.7 ± 1.8 mmHg, and 10.2 ± 1.8 mmHg, respectively. In both groups, the IOP immediately (2 min) after the operation was significantly higher than that at any other time point (*p* < 0.01). IOP values returned to the preoperative baseline level on the first day after surgery, with no significant difference compared with that before injection (*p* > 0.05). IOP continued to be maintained at the preoperative baseline level on the first week after surgery, with no significant difference compared with that before surgery (*p* > 0.05).

**Conclusion:** Infants with ROP who received IVC experienced a sharp increase in the IOP immediately after injection, which decreased to below 30 mmHg after 1 h and maintain that level for 1 week or longer.

## 1 Introduction

Retinopathy of prematurity (ROP) is a leading cause of childhood blindness, it is a vasoproliferative retinal disorder mainly affecting premature infants (Gilbert, 2008; Dogra et al., 2017; Kim et al., 2018). A treatment recommendation for threshold ROP was made by the Multicenter Trial of Cryo-therapy for Retinopathy of Prematurity (CRYO-ROP) (Arch. Ophthalmol., 1988). For the treatment of ROP, laser photocoagulation continues to be the gold standard. Various clinical situations, including poor ocular and systemic conditions, can occur when laser photocoagulation is applied and diminish the outcomes of the treatment. Anti-vascular endothelial growth factor (VEGF) treatment has recently been used for ROP throughout the world (Erol et al., 2015; Yoon et al., 2017).

Many studies exist worldwide on the treatment of ROP using intravitreal injections (IVIs) of bevacizumab and ranibizumab. Discussions about the safety of the treatments have been frequently reported, among which the change in intraocular pressure (IOP) after injections has always been an area of interest. Some studies have shown that the IOP often increases after IVIs of ranibizumab and bevacizumab (Lee et al., 2016; Obata et al., 2019).

Conbercept (KH902; Chengdu Kanghong Biotech Co., Ltd., Sichuan, China) is a recombinant fusion protein that can specifically bind with various isoforms of VEGF-A, VEGF-B, and placental growth factor (PIGF) (Wang et al., 2013). Therefore, conbercept directly interferes with VEGF, which is a vital factor affecting angiogenesis in ROP. The China Food and Drug Administration (CFDA) approved Conbercept for intraocular use in 2013 to treat age-related macular degeneration. Moreover, the administration of multiple conbercept injections for ROP have also been proven to be safe and effective (Jin et al., 2018). The recombinant human IgG1 Fc segment can change the pharmacokinetic characteristics of conbercept and prolong its half-life in circulation, consequently slowing its clearance rate. There are very few reports on the changes in IOP values after intravitreal injection of conbercept (IVC).

This study aimed to explore the changes in the IOP after treatment of ROP in infants using IVC and to supplement the safety studies of conbercept for the treatment of ROP. To the best of our knowledge, this is the first report to evaluate the effect of conbercept on the IOP in the treatment of ROP.

## 2 Materials and methods

### 2.1 Patients

In this prospective study, we investigated premature infants with threshold ROP who received IVC treatment. The International Committee for the Classification of ROP’s revised standards served as the foundation for the definitions of stage and zone (Chiang et al., 2021). Overall, 30 eyes of 15 infants at Guangdong Women and Children Hospital from January 2021 to May 2021. All participants were premature and low-birth-weight infants who underwent examination using the RetCam III wide-angle fundus imaging system and examination of intraocular pressure before surgery. The following were the inclusion criteria: 1) birth weight (BW) < 2500 g, 2) gestational age (GA) < 37 weeks, 3) clinical diagnosis of threshold ROP requiring treatment, and 4) after being fully informed parents of all treated infants about the off-lab l use of IVC for ROP. The exclusion criteria were: 1) previous eye surgery and 2) diseases, including glaucoma, microphthalmia, and other congenital ocular malformations affecting IOP. All patients signed an informed consent form prior to enrollment. This prospective clinical study was approved by the institutional review board and was performed according to the principles of the Declaration of Helsinki.

### 2.2 Injections

Infants were prepared by uniformly applying antibiotic and anesthetic drops before the before the injections, inserting the lid speculum, a 0.1% povidone-iodine (PVP-I) flush for the conjunctival sac, and a 0.9% saline solution flush for the conjunctival sac. The injections were performed using a 30- gauge needle. Each infant received IVC at a dose of 0.25 mg/0.025 mL. In the same session, both eyes received treatment. After injection into the first eye was complete, a new injection procedure for the second eye was initiated with new gloves and instruments. The injection site was between 1.5 and 2 mm posterior to the limbus. While the needle was gently removed, a strabismus hook was applied to stop reflux right away after the injection. All IVC procedures were performed by the same ophthalmologist.

### 2.3 IOP measurements

All pressures were measured using a rebound tonometers (Icare) (TA022, Icare Oy, Vanda, Finland). The IOP was measured before sterile preparation (baseline IOP), immediately after injection (T0), after the first hour (T1), the first day (T2), and the seventh day (T3) following surgery. Further, after excluding the lowest and highest values from the measurement set, the average IOP was provided and the average of three mean IOP values was obtained. The same ophthalmologist performed all IOP measurements, and another ophthalmologist recorded the IOP values. IOP lowering drops would be applied to eyes with an IOP value of 30 mmHg or greater than 30 mmHg for more than 2 h after the injections. All infants underwent fundus examination on the third postoperative day with RetCam 3 to describe and record the retina and optic nerve condition changes (postoperative vs. preoperative).

### 2.4 Statistical analysis

Statistical analysis was performed using the statistical package for the social sciences software version 19.0 (SPSS, Inc., Chicago, IL, United States). The data were analyzed by a one-way ANOVA and paired *t* tests. A *p*-value <0.05 indicates statistical significance.

## 3 Results

We included 30 eyes (15 infants) in this study (Table 1). No serious intraoperative or postoperative complications were observed in any of the cases. All 30 injections of conbercept administered to infants with ROP were half of the adult dose (Table 1). Of the 15 patients, 10 (67%) were males and 5 (33%) were females (Table 2). The mean GA, PMA, and BW of the male group were 28.4 ± 3.0 weeks, 37.1 ± 1.6 weeks, and 1,174 ± 446.0 g, respectively (Table 2). The mean GA, PMA, and BW of the female group were 28.0 ± 2.5 weeks, 36.8 ± 2.1 weeks, and 1,108 ± 285.5 g, respectively (Table 2). As shown in Table 2, no significant differences regarding GA, PMA, and BW were observed between the male group and female group (all *p* > .05, independent samples *t*-test).

**TABLE 1:** Baseline demographics.

Patient	Gender	GA (Weeks + days)	BW(g)	PMA at treatment (weeks + days)	Eye	Dosage (mg)
1	Male	26	880	35 + 4	OD	0.25
					OS	0.25
2	Male	26	850	35 + 4	OD	0.25
					OS	0.25
3	Male	27 + 4	1,000	40 + 1	OD	0.25
					OS	0.25
4	Female	27 + 2	900	36 + 6	OD	0.25
					OS	0.25
5	Female	26 + 3	780	40 + 1	OD	0.25
					OS	0.25
6	Female	25 + 2	1,140	35 + 3	OD	0.25
					OS	0.25
7	Male	26 + 2	990	38 + 6	OD	0.25
					OS	0.25
8	Male	26	2000	37 + 6	OD	0.25
					OS	0.25
9	Male	30 + 5	1,360	35+	OD	0.25
					OS	0.25
10	Male	26 + 1	740	37 + 1	OD	0.25
					OS	0.25
11	female	31	1,510	34 + 6	OD	0.25
					OS	0.25
12	Female	30 + 1	1,210	37 + 5	OD	0.25
					OS	0.25
13	Male	33 + 1	1720	37 + 5	OD	0.25
					OS	0.25
14	Male	25 + 1	700	35 + 5	OD	0.25
					OS	0.25
15	Male	32	1,500	38 + 3	OD	0.25
					OS	0.25

GA, gestational age; BW, birth weight; PMA, postmenstrual age; OD, right eye; OS, left eye.

**TABLE 2:** Baseline characteristics of infants in different sex groups.

Characteristics	Male	Female	*p-*value
Number (%)	10 (67)	5 (33)	—
Mean birth weight ±SD, g	1,174 ± 446.0	1,108 ± 284.6	0.770
Mean gestational age ±SD, week	28.4 ± 3.0	28.0 ± 2.5	0.785
Mean postmenstrual age at injec-tion ±SD, week	37.1 ± 1.6	36.8 ± 2.1	0.773

**FIGURE 1:**
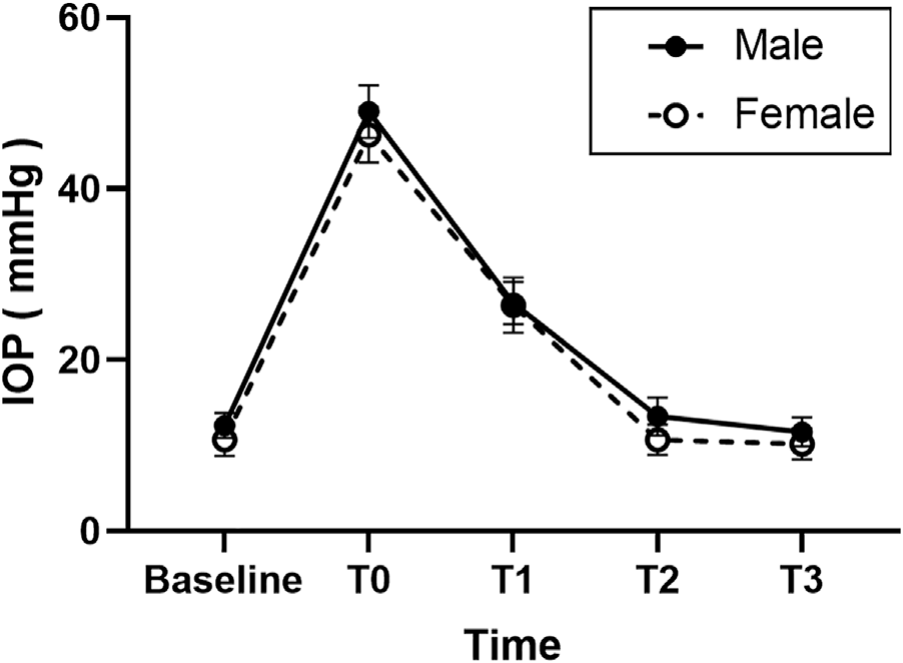
Graph demonstrating the intraocular pressure (IOP) changes after injection of anti-vascular endothelial growth (anti-VEGF) for retinopathy of prematurity (ROP) in different sex groups.

As shown in Figure 1, the mean baseline IOP before injection was higher in the male group (12.4 ± 1.5) than in the female group (10.7 ± 2.0, *p* = 0.016). The mean IOP of T0 was 49.0 ± 3.1 mm Hg in the male group and 47.3 ± 3.2 mm Hg in the female group. The mean IOP of T1 was 26.3 ± 2.5 mm Hg in the male group and 26.4 ± 3.2 mm Hg in the female group. The difference in the mean IOP values at T0 and T1 was not significant in the 2 roups (*p* = 0.053 and *p* = 0.816, respectively). The Mean IOP at T2 and T3 were 13.4 ± 2.2 and 11.6 ± 1.7 mm Hg in the male group and 10.7 ± 1.8 and 10.2 ± 1.8 mm Hg in the female group. As shown in Figure 1, the mean IOP of the male group at T2 and T3 was higher than that in the female group (*p* = 0.002 and *p* = 0.044, respectively).

In both groups, the IOP in all eyes immediately increased sharply from the baseline point at T0 after injection (*p* < 0.01) and decreased to ≤30 mmHg within 1 h (*p* < 0.01); however, it was still higher than the baseline level (*p* < 0.01). There were no significant differences among those three points in all patients (Baseline, T2, and T3) (*p* > 0.05).

## 4 Discussion

Among anti-VEGF drugs used for the treatment of ROP, intravitreous bevacizumab is the most common (Bai et al., 2019; Wallace et al., 2020). Recently, conbercept, a new member of anti-VEGF drug members, has also been used to treat ROP (Bai et al., 2019; Beccasio et al., 2022). However, the risks of using IVC to treat ROP have not been completely explained. IOP increase is an important complication of IVI of razumab and bevacizumab. Conbercept also causes elevated IOP after IVI. Because the IOP of newborns is lower, their tolerance to elevated IOP is also lower than that of adults. We should also consider the safety of IVC while conducting therapies. To our knowledge, only a few studies have reported an IOP increase after anti-VEGF injection in infants (Kato et al., 2019; Obata et al., 2019; Ozdemir et al., 2021). No studies have investigated IOP increase after conbercept injection in infants. However, after anti-VEGF treatments in adult patients, IOP values elevation has been described.

In our study, the IOP values of both male and female infants with threshold ROP increased significantly immediately after IVC. Additionally, in the male group, IOP values of 19 (95%) eyes decreased below 30 mmHg within 1 h, and the other eyes (5%) reduced below 30 mmHg within 2 h. In the female group, the IOP levels of 8 (80%) eyes declined to below 30 mmHg within 1 h, and the other 2 (20%) eyes decreased to below 30 mmHg within 2 h. Moreover, the IOP values of the two groups gradually reduced to baseline levels within 1 day and were maintained for at least 1 week. In this period, there was no obvious trend of repeated increase, suggesting that continuous measurement of IOP may not be required for 2 h after IVC. In other studies, some patients required topical or surgical anti-glaucoma treatments because their IOP increased after IVI (Good et al., 2011; Hoang et al., 2012). Fortunately, for either of the eyes in our investigation, neither anterior chamber paracentesis nor IOP lowering drops was necessary. Additionally, other possible complications of IVI, including infectious endophthalmitis, rhegmatogenous retinal detachment, intraocular hemorrhage, and optic atrophy, were not observed in the infants enrolled in this study. However, we encountered a subconjunctival hemorrhage in two eyes. The subconjunctival hemorrhage was absorbed within 1 week after surgery without treatment. It has been reported that 10% of injections performed on adults resulted in subconjunctival hemorrhage. (Ladas et al., 2009).

A previous study suggested that the change in intraocular volume after IVI plays an important role which causes the elevation of IOP (Morris et al., 2013). The dosage of anti-VEGF drug required for ROP treatment with a single injection remains an ongoing discussion. One report showed that lower doses of bevacizumab (0.375 g) achieved complete regression of intraocular neovascularization in patients with ROP (Harder et al., 2013). In another study, following a 0.025 mL bevacizumab injection for the treatment of ROP, the researchers noticed an increase in IOP after 1 min. They did find a little IOP rise following IVIs in newborn nfan s, though. General anesthesia was used during the measurement process. According to this study, IVI in premature newborns with ROP may not require IOP measurement after all. (Obata et al., 2019). The dose commonly administered in the ROP trial is half the adult dose. In our study, 0.025 mL (0.25 mg) of conbercept was injected. VEGF plays a vital role in organ development, especially in pulmonary alveoli development. VEGF-A and VEGF-B are inhibited by the large protein molecule described a conbercept, which has a molecular weight of 142 kDa. Because of its large protein molecule, it can remain in the vitreous, and the effect lasts longer. Therefore, we thought that the longer time conbercept spent in the vitreous may have caused the longer time to maintain high IOP values than as observed in previous research. Recently, Zhang et al., reported that IOP increased within 2 h after IVC and returned to normal within 24 h without treatment. Conbercept appears to have a safe and long-term effect on patients (Zhang et al., 2022). In our investigation, topical anesthesia with eye drops was used to perform all procedures and assessments. An earlier study found that glaucoma was associated with a higher incidence of psychiatric illnesses (Liu et al., 2020). Pain during surgery may cause emotional agitation and crying in infants, which may cause elevated IOP.

Rushood et al. (2012) found that male individuals had significantly higher central corneal thickness (CCT) than females (Rushood et al., 2012). A study by Uva et al. showed that IOP in premature infants increased with increasing CCT. The authors concluded that the major factor that influences the IOP is CCT (Uva et al., 2011). Another study reported that the IOP measured using Icare was positively correlated with CCT (Pluháček et al., 2019). In the present study, we did not measure CCT in infants. However, we found an increased IOP level in the male group at baseline, T2 and T3, compared with that in the female group, which confirms the report of Rushood et al. We believe that the higher IOP values in the male group may be correlated with higher CCT results. The higher IOP values in the male group than in the female group imply distinct biological aspects between the two sexes. Sex differences depend not only on genetic factors but also on hormonal factors, and these two aspects need to be investigated further.

Some studies showed that an important risk factor for ROP is the male sex (Ying et al., 2015; Slidsborg et al., 2016). In our study group where 15 cases (10 males and females), which were randomly included, showed that the male group was also associated with a higher incidence of ROP.

This study had some limitations. First, the sample size was small, and a single dose of 0.025 mL was used for treatment. Second, the IOP measurements were not taken while the patients were under unified anesthesia, which may have led to bias in the results of this study. Recently, in our clinic, patients who are ineligible for laser therapy due to severe ocular or systemic conditions have received IVIs of anti-VEGF agents. However, a large sample size and further verifications are required in future to affirm our findings.

In conclusion, all 15 infants experienced an increase in the IOP immediately after receiving IVC. Moreover, we observed that all IOP levels normalized to less than 30 mmHg within 2 h after the IVI without any additional treatment and then continued to decrease to baseline levels within 1 day post IVI. Therefore, the danger of increased IOP after IVC treatment for ROP may be time-limited.

## Data Availability

The raw data supporting the conclusion of this article will be made available by the authors, without undue reservation.
